# Supplementary material for: Anti-Tumor Efficacy of In Situ Vaccination Using Bacterial Outer Membrane Vesicles
Source: Cancers (Basel). 2023 Jun 24;15(13):3328. doi: 10.3390/cancers15133328 (PMC10340493; doi:10.3390/cancers15133328)
Supplement: Supplementary file 1 [file cancers-15-03328-s001.zip › cancers-2394516-supplementary.pdf]

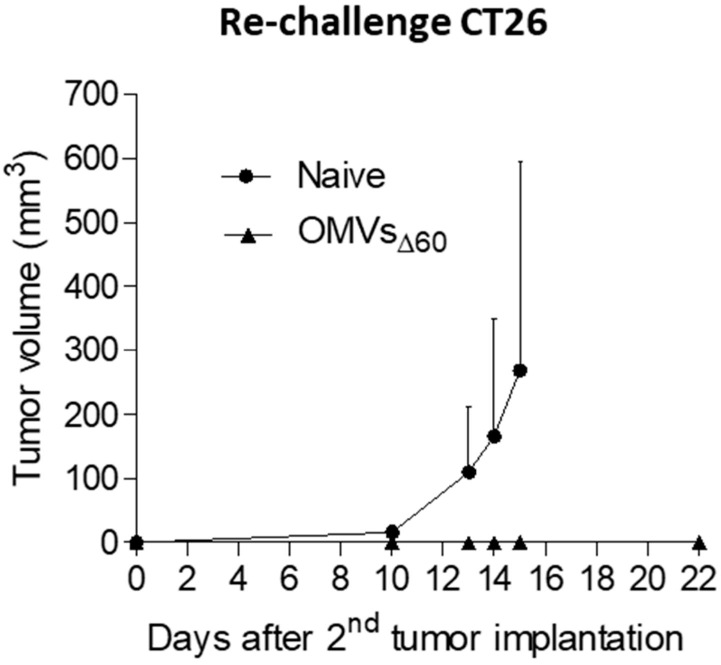

Figure S1. Re-challenge of mice cured after a first OMVs$_{\Delta60}$ ISV treatment – Seven BALB/c mice resulting from previous treatment with OMVs$_{\Delta60}$, with a complete response phenotype were subcutaneously rechallenged 68 days after the first CT26 colon cancer carcinoma tumor cell injection and followed for other 22 days. Five naïve BALB/c mice were used as a positive control for tumor growth. Error bars: mean ± SEM. Naïve n=5, OMVs$_{\Delta60}$ n=7.

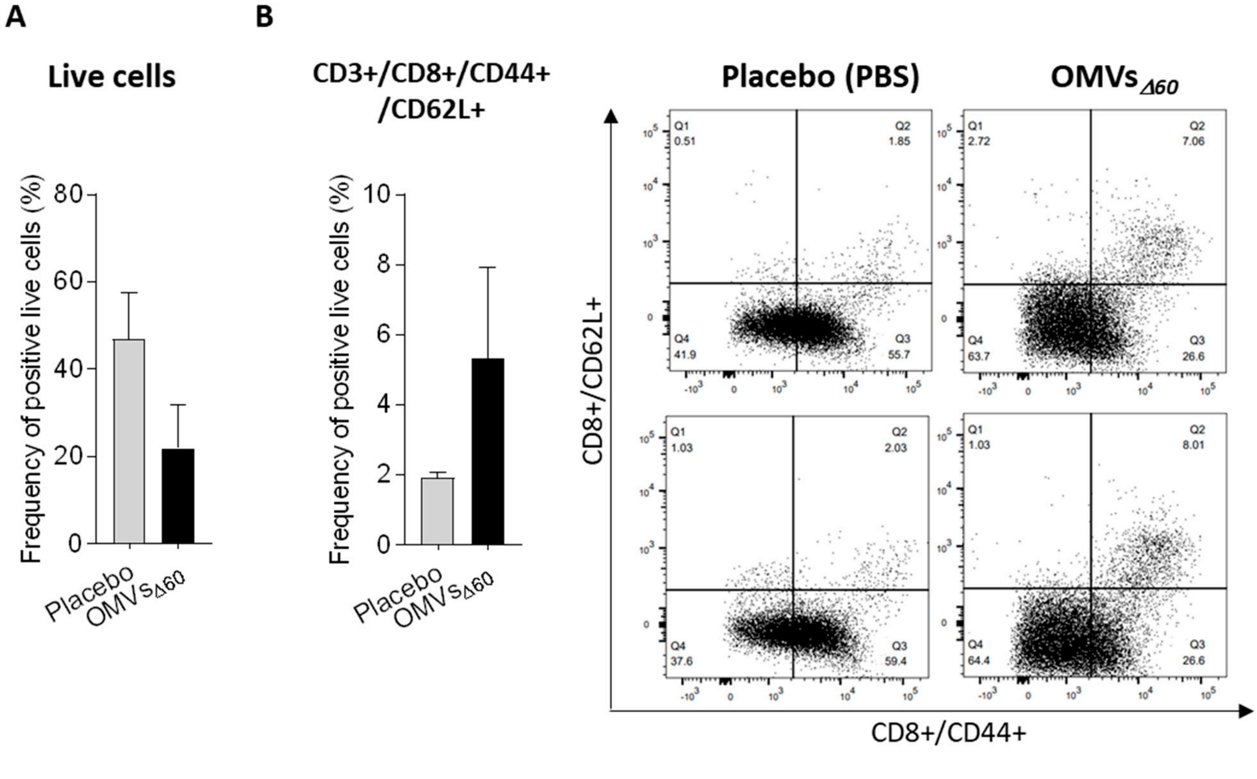

Figure S2. Analysis of cell killing and Central Memory CD8+ T cells in tumor by flow cytometry – BALB/c mice were immunized intratumorally three times with PBS or 1 µg of OMVs$_{\Delta60}$ two days apart. 24h after the third treatment tumors were collected and analyzed by flow cytometry. The figure reports the flow cytometry analysis of the frequency of live cells in tumors treated with either

PBS (n=2) or with OMVs$_{\Delta 60}$ (n=4), data are reported as bar graphs (A). (B) Frequency of tumor infiltrating CD8+/CD44+/CD62L+ Central Memory CM) T cells in tumors treated with either PBS (n=2) or with OMVs$_{\Delta 60}$ (n=4), data are reported both as bar graphs and dot plots. .
